# Performance characteristics of highly automated HSV-1 and HSV-2 IgG testing

**DOI:** 10.1128/jcm.00263-24

**Published:** 2024-04-30

**Authors:** Katharine H. D. Crawford, Stacy Selke, Gregory Pepper, Erin Goecker, Aniela Sobel, Anna Wald, Christine Johnston, Alexander L. Greninger

**Affiliations:** 1 Department of Laboratory Medicine and Pathology, University of Washington, Seattle, Washington, USA; 2 Department of Medicine, University of Washington, Seattle, Washington, USA; 3 Department of Epidemiology, University of Washington, Seattle, Washington, USA; 4 Vaccine and Infectious Disease Division, Fred Hutchinson Cancer Center, Seattle, Washington, USA; Mayo Clinic Minnesota, Rochester, Minnesota, USA

**Keywords:** HSV, HSV-1, HSV-2, IgG, serology, Roche, Diasorin, Bio-Rad, western blot, herpes

## Abstract

Herpes simplex virus (HSV) infections are one of the most common and stigmatized infections of humankind, affecting more than 4 billion people around the world and more than 100 million Americans. Yet, most people do not know their infection status, and antibody testing is not recommended, partly due to poor test performance. Here, we compared the test performance of the Roche Elecsys HSV-1 IgG and HSV-2 IgG, DiaSorin LIAISON HSV-1/2 IgG, and Bio-Rad BioPlex 2200 HSV-1 and HSV-2 IgG assays with the gold-standard HSV western blot in 1,994 persons, including 1,017 persons with PCR or culture-confirmed HSV-1 and/or HSV-2 infection. Across all samples, the Bio-Rad and Roche assays had similar performance metrics with low sensitivity (<85%) but high specificity (>97%) for detecting HSV-1 IgG and both high sensitivity (>97%) and high specificity (>98%) for detecting HSV-2 IgG. The DiaSorin assay had a higher sensitivity (92.1%) but much lower specificity (88.7%) for detecting HSV-1 IgG and comparatively poor sensitivity (94.5%) and specificity (94.2%) for detecting HSV-2 IgG. The DiaSorin assay performed poorly at low-positive index values with 60.9% of DiaSorin HSV-1 results and 20.8% of DiaSorin HSV-2 results with positive index values <3.0 yielding false positive results. Based on an estimated HSV-2 seroprevalence of 12% in the United States, positive predictive values for HSV-2 IgG were 96.1% for Roche, 87.4% for Bio-Rad, and 69.0% for DiaSorin, meaning nearly one of every three positive DiaSorin HSV-2 IgG results would be falsely positive. Further development in HSV antibody diagnostics is needed to provide appropriate patient care.

**IMPORTANCE:**

Serological screening for HSV infections is currently not recommended in part due to the poor performance metrics of widely used commercial HSV-1 and HSV-2 IgG assays. Here, we compare three Food and Drug Administration (FDA)-cleared automated HSV-1 and HSV-2 IgG assays to the gold-standard western blot across nearly 2,000 samples. We find that not all commercially available HSV assays are created equal, with comparably low sensitivities for HSV-1 IgG across platforms and high false positivity rates for DiaSorin on HSV-2 IgG. This study is the first large-scale comparison of performance metrics for the Bio-Rad and Roche assays in over 10 years. Our study confirms that there remains room for improvement in HSV serological diagnostic testing—especially in regard to low sensitivities for HSV-1 IgG detection—and highlights that some previously less-studied assays may have better performance metrics than previously considered typical of commercially available HSV-2 IgG assays.

## INTRODUCTION

Herpes simplex virus types 1 and 2 (HSV-1 and HSV-2) are important human pathogens that cause lifelong infections characterized by latent infection with cycles of viral mucosal replication. Classically, HSV infections present with episodes of recurrent, painful lesions of the oral or genital epithelium, but many infected persons remain asymptomatic (1). HSV is characterized by two types: HSV-1 is near-ubiquitous and infects an estimated 67% of the world’s population and ~48% of adults in the United States of America (2, 3). HSV-1 is associated with oral lesions but is an increasingly recognized cause of genital herpes (4). Conversely, HSV-2 is classically associated with genital herpes and infects approximately 13% of adults worldwide and ~12% of adults in the United States of America (2, 3).

HSV infections can be diagnosed by detecting the virus itself—typically through culture or PCR—or detecting antibodies against viral proteins (5). The HSV lifecycle, which includes cycles of lytic and latent infection, complicates diagnosis and limits accurate PCR testing to episodes of active mucosal infection. Not all episodes of active mucosal infection are symptomatic, and high rates of asymptomatic shedding mean symptoms alone cannot be used to guide testing to prevent transmission (6). High-throughput, sensitive, and specific serological diagnostics are required for both clinical care and for potential screening to diagnose the vast majority of HSV infections that currently go undetected (7).

Most high-throughput HSV serology assays detect type-specific IgG responses to the HSV-1 or HSV-2 glycoprotein G (gG_1_ for HSV-1 and gG_2_ for HSV-2), which share only ~40% amino acid identity (8). However, IgG to gG_1_ and gG_2_ may still be cross-reactive and insufficiently specific, leading the United States Preventive Task Force to recommend against screening for genital herpes infections among asymptomatic adolescents and adults (9). Centers for Disease Control and Prevention (CDC) Sexually Transmitted Infection (STI) Treatment Guidelines recommend a two-step testing process, using a sensitive enzyme/chemiluminescence assay for screening followed by a second, more specific confirmatory assay (10). The gold standard for confirmatory serological diagnosis of HSV-1 and HSV-2 infections is a western blot (WB) that detects serological responses to multiple proteins within a viral lysate (11). This assay is highly sensitive and specific, but it is laborious and thus costly and only available in the United States at one reference lab. Here, we examined the sensitivity and specificity of three fully automated, high-throughput, type-specific HSV-1/2 IgG assays compared to the HSV western blot in two clinical cohorts comprising 1,994 individuals.

## MATERIALS AND METHODS

### Cohorts

#### HSV western blot serum specimen remnant cohort

The initial cohort consisted of samples selected from the University of Washington (UW) HSV WB sample remnants collected from January 2022 through April 2023. Sample selection was restricted to the 5,423 samples collected within the UW Medicine system to ensure the availability of clinical metadata, to approximate a more typical HSV testing population (since all UW specimens are tested only by WB without a prior screening test), and to avoid biasing specimens toward indeterminate results sent to our reference lab for WB confirmatory testing. Based on the WB results, we randomly selected 449 samples that were negative for both HSV-1 and HSV-2 antibodies, 177 samples that were positive for HSV-1 and negative for HSV-2 antibodies, 182 samples that were positive for HSV-2 and negative for HSV-1 antibodies, and 184 samples that were positive for both HSV-1 and HSV-2 antibodies. For each WB category, each sample came from a different person. Two persons contributed two samples as their WB serological status changed during the sample selection timeframe, and their samples were randomly selected in two result categories. Due to low sample volume, 13 of the originally selected samples were unable to be tested leaving 979 samples from 977 persons. Additionally, due to low sample volume and rare barcode errors, not all samples were able to be run on all instruments. The number of samples from this cohort runs on each instrument and their concordance with the WB result are presented in Table 1. The use of excess clinical testing specimens was approved by the UW Institutional Review Board with a consent waiver (STUDY00010205).

**TABLE 1:** Number of samples from the clinical testing remnant sample cohort that were tested on each automated instrument per WB category^*a*^

WB category	Number tested: all assays	Number tested: Bio-Rad BioPlex 2200 HSV-1 and HSV-2 IgG	% Concordance: Bio-Rad		Number tested: DiaSorin LIAISON HSV-1 and HSV-2 IgG	% Concordance: DiaSorin		Number tested: Roche Elecsys HSV-1 IgG and HSV-2 IgG	% Concordance: Roche	
			HSV-1	HSV-2		HSV-1	HSV-2		HSV-1	HSV-2
HSV-1 neg and HSV-2 neg	444	434	99.3%	99.5%	443	91.4%	94.8%	435 and 427	100%	100%
HSV-1 pos and HSV-2 neg	175	172	88.4%	96.5%	175	93.1%	92.0%	173 and 171	86.1%	98.8%
HSV-1 neg and HSV-2 pos	182	181	95.6%	93.4%	182	82.4%	91.2%	169 and 165	95.3%	97.0%
HSV-1 pos and HSV-2 pos	178	178	85.4%	88.8%	178	94.4%	78.1%	168 and 168	85.7%	95.2%
Total	979	965	94.1%	95.9%	978	90.6%	90.6%	945 and 931	94.1%	98.4%

^*a*^ Some samples were unable to be run on all instruments due to different volume requirements or rare barcode errors. The percent concordance with the WB results for each assay is also shown. For the Roche HSV-1 and HSV-2 assays, sample numbers are provided for each assay (e.g., HSV-1 sample number and HSV-2 sample number) since some samples had insufficient volume to be run on both assays.

#### PCR- or culture-confirmed cohort

A second cohort consisted of serum specimens from persons with genital lesions PCR- or culture-positive for HSV-1 or HSV-2 followed by the UW Virology Research Clinic who provided written informed consent approved by the UW Institutional Review Board (IRB) (12–14). We selected one serum specimen each from 1,074 HIV-negative persons who tested positive for HSV-1 or HSV-2 by PCR and/or culture and were confirmed seropositive for the corresponding HSV type by WB. Samples were collected at least 6 months after a person’s initial episode of genital herpes. Some persons with HSV-2 positive genital lesions had both HSV-2 and HSV-1 antibodies when tested with the WB, resulting in 131 samples positive for HSV-1 only with both viral and WB testing, 566 samples positive for HSV-2 only with both viral and WB testing, and 320 samples positive for HSV-2 with viral testing and both HSV-1 and HSV-2 antibodies on WB. Virological data on shedding rates in genital herpes infections (measured as percent of swabs positive for HSV-1 or HSV-2 by non-type-specific PCR over total number of swabs) were also available from 315 of 1,017 persons in the Virology Research Clinic (VRC) cohort (shedding rates available in Supplemental Data file) (15).

### Assay comparison

The DiaSorin Liaison HSV-1 and HSV-2 IgG chemiluminescent immunoassays (CLIAs; K081685 and K081687; DiaSorin, Stillwater, Minnesota, USA), the Roche cobas Elecsys HSV-1 and HSV-2 IgG sandwich electrochemiluminescence immunoassays (K120625 and K121895; Roche, Indianapolis, Indiana, USA), and the Bio-Rad BioPlex HSV-1/2 IgG multiplexed bead immunoassay (K120959; Bio-Rad, Hercules, California, USA) were compared to the HSV-1 and HSV-2 WB performed at the UW Virology lab. These commercially manufactured assays are widely used fully automated HSV serology assays that are FDA 510(k) cleared for clinical use in the United States. Quality control (QC) and calibration were performed for each assay according to laboratory procedures for HSV WB or the manufacturer’s instructions for commercial assays.

#### Western blot

All samples were previously tested for HSV-1 and HSV-2 antibodies by WB at UW as part of clinical testing or research (11). In this assay, HSV-1 and HSV-2 proteins from lysates of HSV-infected cells are separated by gel electrophoresis and then transferred to nitrocellulose paper to make HSV-type-specific blots containing separated, fixed proteins from either HSV-1 or HSV-2. These blots are then incubated with a 1:50 dilution of the patient’s serum, and antibodies that bind the viral proteins are detected using peroxidase-labeled goat anti-human IgG antibodies and a subsequent enzyme-mediated color change. The staining pattern of the antibodies in the patient’s serum on each blot allows for type-specific determination of HSV antibodies and determination of a patient’s serological status. Blots are read by three independent readers, and reader consensus is used to call samples positive, negative, or indeterminate for HSV-1 and HSV-2 antibodies based on the presence or absence of a typical banding pattern (11). Notably, if any reader finds the initial blot indeterminate due to an atypical banding pattern or very faint bands, samples are adsorbed for HSV-1 and HSV-2 antibodies and re-run or repeated with more sample volume (11). This process improves both the sensitivity and specificity of the WB assay by improving the ability of the assay to distinguish true reactivity from cross-reactive antibodies and improving the detection of samples with faint banding patterns (11). Following adsorbing or repeating the sample, results are re-read by three independent readers and consensus-called as positive, negative, or indeterminate. No samples with a final WB result of indeterminate were included in this study. Following initial clinical or research testing, samples were stored at −10°C to −25°C until use in this study (average of 12 months for clinical testing remnants or ~25 years for the VRC samples). Most samples underwent two freeze/thaw cycles for this study—one for testing on the DiaSorin and Roche instruments and one for testing on the Bio-Rad instrument. Some samples that were initially equivocal on the DiaSorin or Bio-Rad assays underwent a third freeze/thaw cycle as they were repeated in duplicate after the initial testing was completed.

#### DiaSorin LIAISON HSV-1- and HSV-2-type-specific IgG assays

Samples were thawed and briefly mixed prior to testing with the DiaSorin LIAISON HSV-1 and HSV-2 IgG CLIA according to the manufacturer’s instructions. In this assay, magnetic beads coated with recombinant antigens specific for either HSV-1 glycoprotein (gG1) or HSV-2 glycoprotein (gG2) are incubated with patient serum. After washing, the beads are incubated with an isoluminol-conjugated anti-human IgG mouse monoclonal antibody. Another wash removes any unbound antibody prior to inducing a flash chemiluminescence reaction. The resulting light signal, measured as relative light units (RLUs), indicates how much isoluminol-antibody conjugate and, thus, HSV-1 or HSV-2 IgG is present in the sample. The RLUs are converted to an index value using the on-board calculation based on the manufacturer-provided calibrators. This index value is used to categorize samples as negative (index *<* 0.9), equivocal (index 0.90–1.09), or positive (index ≥ 1.1). No reportable range is listed on the package insert. The reported index values in this study ranged from <0.01 to >62.2 for the DiaSorin HSV-1 assay and from 0.03 to >23.6 for the HSV-2 assay. Linearity was confirmed for index values between 0.1 and 47 for HSV-1 and 0.15 and 21 for HSV-2 (Fig. S1; Supplemental Data 2). Samples that were initially reported as equivocal were repeated at least twice, and the median measurement was reported as the final result. For the VRC cohort, the 34 initially equivocal samples were repeated at the end of the study, following an additional freeze/thaw. Following testing on the DiaSorin instrument, samples were stored at 4°C for *<*48 hours prior to testing using the Roche Elecsys assays.

#### Roche Elecsys HSV-1 IgG and HSV-2 IgG assays

All samples were tested using the Roche Elecsys HSV-1 and HSV-2 IgG immunoassays according to the manufacturer’s instructions. In this assay, serum samples are incubated with biotinylated recombinant HSV-1 gG1 or HSV-2 gG2 antigens, and the same antigens are labeled with a ruthenium complex to form a double-antigen sandwich complex. Streptavidin-coated magnetic microparticles are then added, and the sandwich complex binds to this solid phase through the interaction of biotin and streptavidin. The microparticles are then magnetically captured on the surface of an electrode, and all unbound substances are washed away. Applying a voltage to the electrode induces chemiluminescence of the ruthenium complex which is measured by a photomultiplier. On-board software converts this electrochemiluminescent signal to a cutoff index (COI) based on the manufacturer-supplied calibrators. A COI *<* 1.0 is considered non-reactive (negative), and a COI ≥ 1.0 is reactive (positive), with no equivocal range. Neither low nor high values are censored as > or < and reported values ranged from a COI of 0.026–234.8 for HSV-1 and 0.083–575.1 for HSV-2. Linearity was confirmed from 0.1 to 140 for HSV-1 and 0.2–350 for HSV-2 (Fig. S1; Supplemental Data 2). After testing using the Roche Elecsys assays, samples were frozen at −20°C for 2–3 weeks prior to running on the Bio-Rad BioPlex instrument.

#### Bio-Rad BioPlex 2200 HSV-1 and HSV-2 IgG assay

Serum samples were thawed, mixed, and briefly spun (700 × *g* for 4 min) prior to testing with the Bio-Rad BioPlex 2200 HSV-1 and HSV-2 IgG assay according to the manufacturer’s instructions. The BioPlex assay is a multiplexed microparticle immunoassay. Briefly, serum samples are incubated with dyed beads coated with either recombinant HSV-1 gG1 antigen or a synthetic HSV-2 gG2 peptide. After washing, anti-human IgG antibody conjugated to phycoerythrin is added, and the specimens are incubated at 37°C. After another wash cycle, the mixture passes through a detector that measures the fluorescence of both the bead dye and phycoerythrin. This allows for simultaneous determination of antibody amount and antigen type (e.g., HSV-1 or HSV-2). Relative fluorescence intensity values are converted to antibody index (AI) using the on-board software and manufacturer-provided calibrators. An AI of *<*0.9 is negative, 0.9–1.0 is equivocal, and ≥1.1 is positive. Results with indices less than 0.2 or greater than 8.0 are reported as <0.2 or >8.0, respectively. With this narrow measurement range, only 31% of results in this study had a non-bounded numerical result (i.e., from 0.2 to 8.0). Linearity was confirmed across the measurement range (0.2–8.0 AI; Fig. S1; Supplemental Data 2). Bio-Rad recommends obtaining an additional sample and repeating testing for patients with equivocal results. We could not test additional samples but did repeat all equivocal specimens at least twice to assess the reproducibility of these equivocal results. Of the 35 initially equivocal samples, 25 were repeated after an additional freeze-thaw cycle. For repeated samples, the median result was accepted as the final result.

## RESULTS

### Clinical testing remnant samples cohort

#### Samples and demographics

HSV-1 and HSV-2 IgG were measured in 979 remnant WB samples from 977 individuals from all four WB result categories: 444 negative for both HSV-1 and HSV-2 IgG, 175 positive for HSV-1 but negative for HSV-2 IgG, 182 positive for HSV-2 IgG but negative for HSV-1 IgG, and 178 positive for IgG to both HSV-1 and HSV-2. For each instrument, we calculated concordance with the WB results (Table 1).

The clinical remnant samples consisted of 474 (49%) women and 503 (51%) men with a median age of 43 years (range: 3–91 years; Table S1). The age distribution was bimodal, which was likely related to the two separate major indications for HSV serology testing—STI testing and pre-transplant screening (Fig. S2; Table S1). Most people identified as White and non-Hispanic (64.3%) and were from Washington state (87.1%).

#### Comparison to WB for clinical testing sample remnants

No single assay performed best for both HSV-1 and HSV-2 in the clinical remnant cohort. The DiaSorin HSV-1 IgG assay had the highest sensitivity (94.8%) but lowest specificity (90.4%), while both the Roche and Bio-Rad assays had lower sensitivities (85.9% and 87.1%, respectively) but higher specificities (98.7% and 98.2%, respectively; Table 2). For measuring HSV-2 IgG, the Roche assay had the highest sensitivity (96.1%) and specificity (99.7%). The Bio-Rad HSV-2 IgG assay had a sensitivity of 91.9% and specificity of 98.8%, and the DiaSorin HSV-2 IgG assay was both the least sensitive (85.4%) and least specific (94.5%).

**TABLE 2:** Test results for each automated instrument compared to the WB results for the clinical testing remnant sample cohort^*a*^

		HSV-1 IgG								HSV-2 IgG							
		Bio-Rad *n* = 965			DiaSorin *n* = 978			Roche *n* = 945		Bio-Rad *n* = 965			DiaSorin *n* = 978			Roche *n* = 931	
		Pos	Neg	Eqv	Pos	Neg	Eqv	Pos	Neg	Pos	Neg	Eqv	Pos	Neg	Eqv	Pos	Neg
Western blot	Pos	304	45	1	331	18	4	293	48	327	29	3	305	52	3	320	13
	Neg	11	604	0	59	555	11	8	596	7	598	1	34	581	3	2	596
Sensitivity (95% CI)		87.1% (83.1%–90.4%)			94.8% (92.0%–96.9%)			85.9% (81.8%–89.4%)		91.9% (88.5%–94.5%)			85.4% (81.3%–88.9%)			96.1% (93.4%–97.9%)	
Specificity (95% CI)		98.2% (96.8%–99.1%)			90.4% (87.8%–92.6%)			98.7% (97.4%–99.4%)		98.8% (97.6%–99.5%)			94.5% (92.4%–96.1%)			99.7% (98.8%–100%)	

^*a*^ Equivocal (Eqv) results were not included in calculating sensitivity and specificity.

On initial testing, 19 results (1.0%; 8 HSV-1 and 11 HSV-2) were equivocal on the Bio-Rad assay, and 24 results (1.2%; 16 HSV-1 and 8 HSV-2) were equivocal on the DiaSorin assay. For the Bio-Rad assay, the majority of equivocal results—88% for HSV-1 and 64% for HSV-2—were revised to a non-equivocal final result of which 57% of the revised results were concordant with WB. Only 12.5% of the DiaSorin equivocal results had a non-equivocal final result, but all revised results were concordant with WB (see Supplemental Material for detailed discussion). Only two samples were equivocal on more than one assay. One sample was HSV-1 IgG positive only by WB and had initially equivocal results on the Bio-Rad HSV-1 IgG assay and the DiaSorin HSV-2 IgG assay. After repeating, this sample was correctly called positive for HSV-1 IgG on the Bio-Rad instrument but remained equivocal for HSV-2 IgG on the DiaSorin instrument. The other sample was positive for both HSV-1 and HSV-2 IgG by WB but equivocal for both analytes on the DiaSorin instrument and remained equivocal after repeating. Overall, equivocal results were more platform specific than sample specific as there were no samples that were equivocal for the same analyte on multiple instruments.

#### Investigation of discordant results

**Fig 1:**
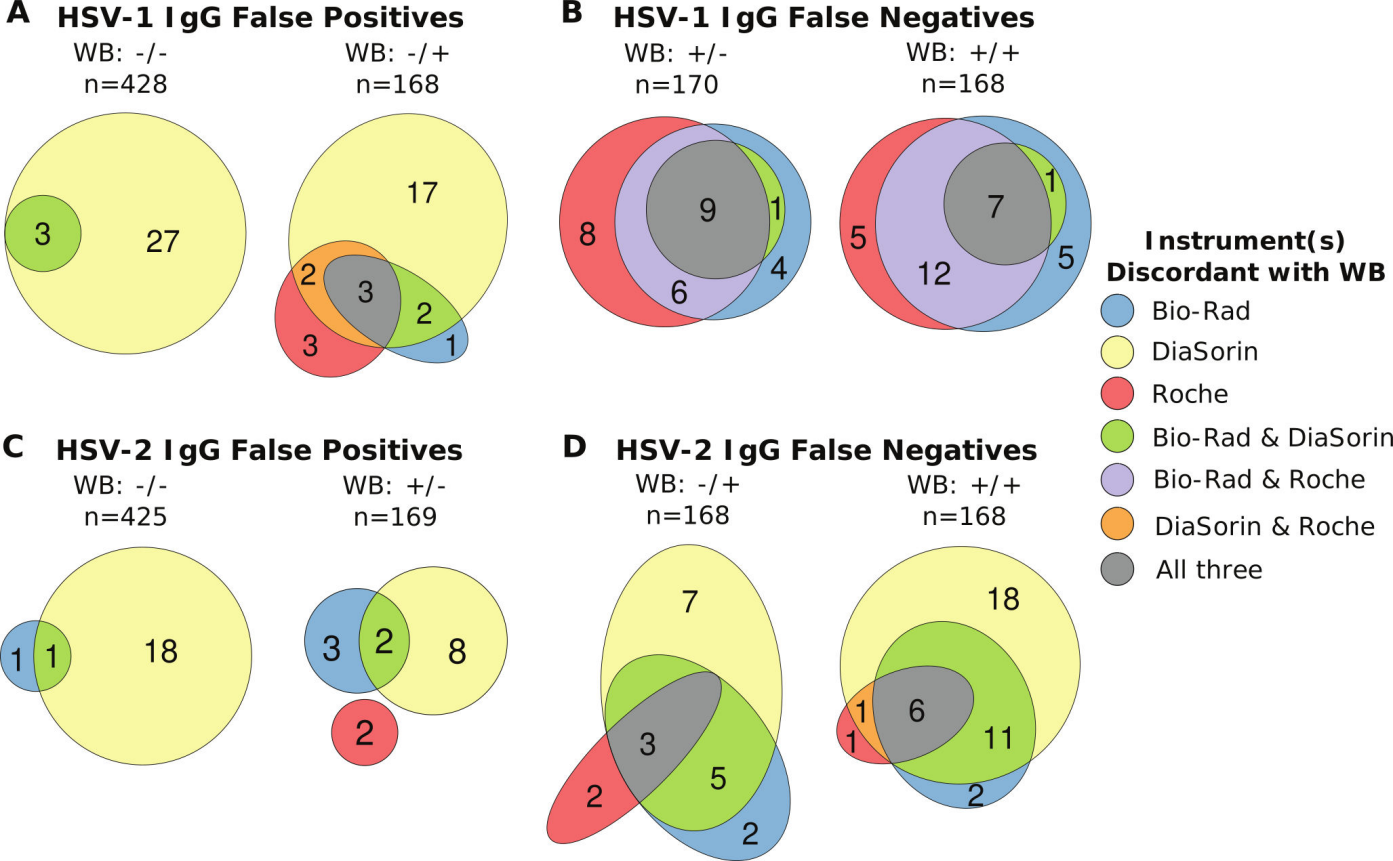
Euler plots showing the number of samples that disagree with the WB results for each automated instrument colored by which instruments were discordant with the WB results. Data are presented separately for each WB category with the HSV-1 WB result left of the “/” and the HSV-2 WB result right of the slash. Only samples that were run on all three instruments are included with *n* showing the number of samples tested on all three instruments for the given WB category. Within each category, the size of each colored section is roughly proportional to the number of samples contained within it. (**A**) Number of samples that were negative for HSV-1 IgG by WB but falsely positive on the automated instruments. (**B**) Number of samples that were positive for HSV-1 IgG by WB but falsely negative on the automated instruments. (**C**) Number of samples that were negative for HSV-2 IgG by WB but falsely positive on the automated instruments. (**D**) Number of samples that were positive for HSV-2 IgG by WB but falsely negative on the automated instruments. Plots made using the eulerr R package (16).

We next examined whether the same specimens had discordant results compared to WB on multiple automated instruments. In total, 201 of the 997 samples in the clinical testing sample remnant cohort (20.5%) had results discordant with the WB result on at least one instrument for at least one analyte (e.g., HSV-1 IgG or HSV-2 IgG). Of these 201 samples, 102 (51%) were discordant for HSV-1 only, 78 (39%) were discordant for HSV-2 only, and 21 (10%) were discordant for both assays on at least one instrument. Since 21 samples were discordant with the WB results for both HSV-1 and HSV-2, in total 222 assay, results were discordant with the WB results on at least one automated instrument. Notably, 13 of these results came from samples that were not run on all three instruments (Table 1). Excluding those, 134 results were discordant on one instrument only, 47 were discordant on two tests, and 28 were discordant with the WB results on all three tests (Fig. 1).

We reviewed the original WB blots for 25 of the samples that were discordant with the WB on two or three automated assays. These samples were selected to represent all four possible discordant situations (e.g., HSV-1 IgG positive by WB, but negative by the automated assays, etc.), and 12 of these samples were originally adsorbed or repeated during clinical testing. For this review, independent readers were aware that there was a discordance but blinded to what assays were discordant. On review, no readers would have changed the final interpretation of any blots, but there were three samples—two for HSV-2 and one for HSV-1—that were positive on the WB and negative on all three automated assays where at least one reader would now recommend adsorbing the sample for further clarification. There were only three samples where all three automated instruments called the sample positive for HSV antibodies, and the WB was negative. All three samples were discordant for HSV-1 IgG, and the blot reviewers noted that the HSV-1 blot was more consistent with HSV-1 cross-reactivity than true HSV-1 IgG. Overall, a review of the original WBs for a subset of samples where multiple automated assays were discordant with the WB found that 3 out of 25 samples would now be recommended for further testing (e.g., adsorption), but the expert readers agreed with the original clinical WB result in the vast majority of cases (88%). For this assay comparison study, the WB results were considered the ground truth even for samples where more than one assay was discordant with the WB.

We found that the false positivity rate for HSV-1 or HSV-2 antibodies was higher in samples that were positive for antibodies to the other virus. For HSV-1, 17% (31 of 182) of samples that were seropositive for HSV-2 only by WB tested falsely positive for HSV-1 IgG on at least one automated instrument compared with 7.4% (33 of 444) of samples that were negative for any HSV antibodies by WB (two sample *z* test: *P*-value < 0.001). There was a similar trend for HSV-2 as 9.7% (17 of 175) of samples that were positive for HSV-1 only by WB tested falsely positive for HSV-2 IgG on at least one automated instrument, while only 5.2% (23 of 443) of samples that were negative for all HSV antibodies by WB tested falsely positive for HSV-2 IgG (two sample *z* test: *P*-value = 0.06). Notably, 76% of HSV-1 false positive and 74% of HSV-2 false positive test results came from the DiaSorin instrument. For HSV-1, the DiaSorin instrument had fewer false negative results than the other instruments, and there were no samples where it was the only instrument to falsely call a sample negative (Fig. 1). However, for HSV-2, the DiaSorin instrument had numerous false negative results, and 43% (25 of 58) of the HSV-2 false negative results were unique to the DiaSorin instrument.

During initial clinical WB testing, 168 of the 979 (17.2%*)* samples were repeated with a larger sample volume or adsorbed for HSV-1 and HSV-2 antibodies and re-tested. These samples that required repeating or absorption were significantly more likely to give discordant results on at least one automated assay (two-sample *z* test: *P*-value < 0.001). Of the 168 samples that were absorbed or repeated, 46% disagreed with the WB results on at least one automated assay. In contrast, among the 811 samples that were not adsorbed or repeated only 15% of samples had a discordant result on at least one automated assay.

#### Semi-quantitative results

**Fig 2:**
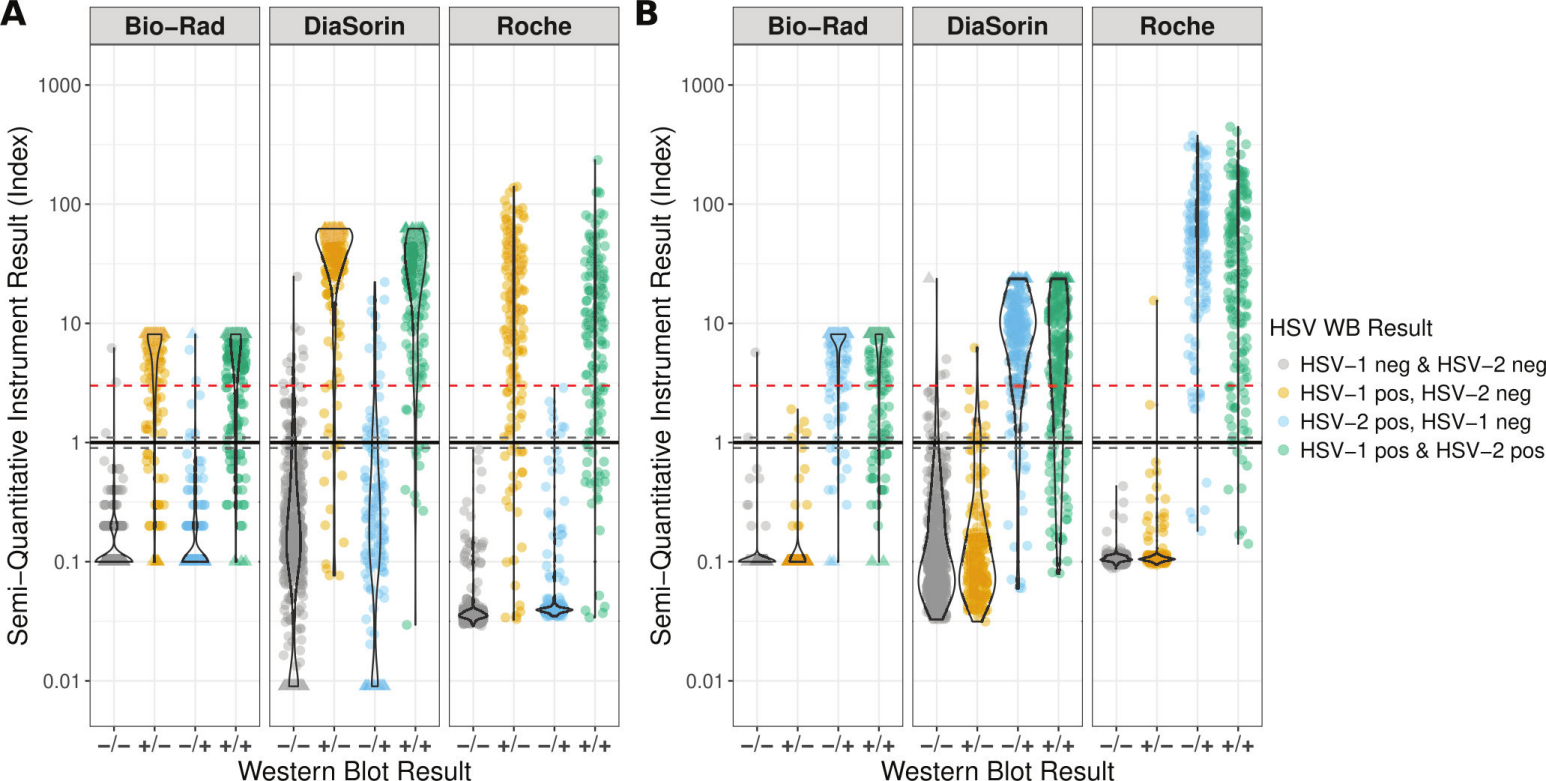
Comparison of semi-quantitative assay results for each instrument and known WB category for the clinical testing sample remnant cohort. (**A**) HSV-1 IgG test results. (**B**) HSV-2 IgG test results. The black line shows the cutoff value of 1.0 with the gray dotted lines at 0.9 and 1.1 delineating the index values that are called equivocal by the Bio-Rad and DiaSorin instruments. The red dotted line at 3.0 marks a common cutoff for “low-positive” results. Values outside the reportable range for the Bio-Rad and DiaSorin instruments were assigned values 0.1 above or below the cutoff value and are shown as triangles.

**Fig 3:**
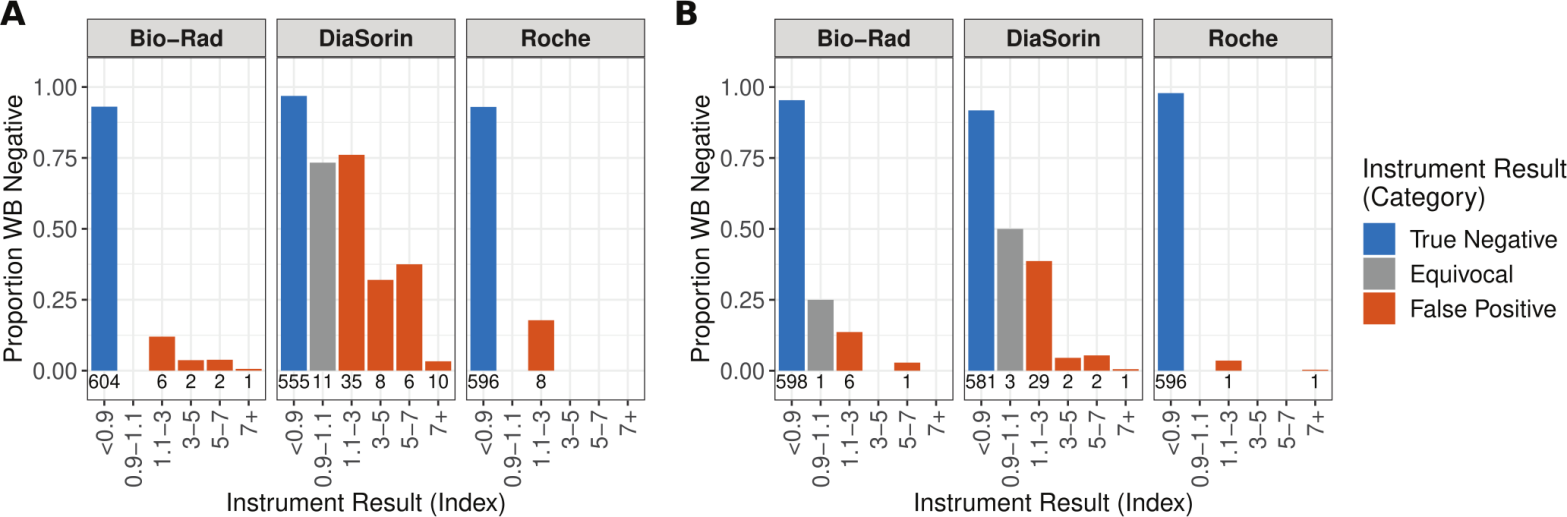
Percent of results that are negative by WB for each index value bin for each instrument for the clinical testing remnant sample cohort. (**A**) HSV-1 IgG. (**B**) HSV-2 IgG. All samples to the right of 1.1 are false positive results for the automated analyzer. The number below each bar is the number of samples represented by the bar.

Although each assay’s primary result is positive, negative, or equivocal (for the Bio-Rad and DiaSorin assays) for HSV-1 or HSV-2 IgG, each automated assay also provides a semi-quantitative index value. Previous studies have found that “low-positive” index values of <3.0 (Fig. 2, red line) are associated with more false positive results and a decreased positive predictive value (14). Indeed, when samples are grouped by index value, samples with positive index values <3.0 have the highest percent of false positive results for both HSV-1 and HSV-2 IgG on all instruments (Fig. 3). This is especially true for the DiaSorin instrument where 76.1% of low-positive index values are falsely positive for HSV-1 IgG, and 38.7% of low-positive results are falsely positive for HSV-2 IgG.

### VRC cohort

#### Samples and demographics

To further examine automated HSV serology assay performance, we took advantage of a unique cohort of 1,017 persons with genital HSV infection confirmed by PCR or culture through genital herpes studies at the UW VRC from 1981 to 2019. For this study, we included samples from persons with genital herpes with PCR- or culture-confirmed HSV infection who had serological testing using the WB at least 6 months (median 5.8 years, range 6 months to 50 years) after the initial episode of genital herpes. Given the focus on genital herpes, 886 (87%) people in this cohort had PCR- or culture-confirmed HSV-2 infections, and 131 (13%) people had PCR- or culture-confirmed genital HSV-1 infections. WB results were 100% concordant with the PCR or culture results. Of the 886 people with confirmed HSV-2 genital infections, 320 (36%) also had HSV-1 antibodies detected on the WB, most likely from prior HSV-1 infection.

The VRC cohort was composed of 669 women (65.8%) and 348 men (34.2%) who provided samples between 1981 and 2019 (median 1989). Most people were white (86.3%), and the overall median age was 34.3 (range: 16–88 years, Table S2; Fig. S2).

#### Assay performance

The VRC cohort comprises samples that are positive for HSV-1 or HSV-2 antibodies based both on WB results and viral confirmation of genital infection. In this cohort, all automated instruments had lower sensitivity and specificity for detecting HSV-1 IgG with all instruments having a sensitivity <90%. Conversely, in this population, the automated assays are more sensitive for detecting HSV-2 antibodies than in a typical screening population (Table 3). However, the automated assays had lower specificity for HSV-2 IgG in this cohort. When only analyzing samples with PCR- or culture-confirmed HSV-1 or HSV-2 genital infection [e.g., only positive for either HSV-1 IgG (131 samples) *or* HSV-2 IgG (566 samples)], the sensitivities of the HSV-1 IgG assays were not improved (80.6% for Bio-Rad, 87.8% for DiaSorin, and 69.5% for Roche). For HSV-2, all instruments’ sensitivities were largely unchanged and were within 0.6% of their values from the full cohort. Assay specificities for this subset were unchanged compared to the full cohort (Table S3).

**TABLE 3:** Test results for each automated instrument compared to the WB results for the VRC cohort^*a*^

		HSV-1 IgG								HSV-2 IgG							
		Bio-Rad *n* = 1,001			DiaSorin *n* = 1,012			Roche *n* = 1,000		Bio-Rad *n* = 1,001			DiaSorin *n* = 1,015			Roche *n* = 996	
		Pos	Neg	Eqv	Pos	Neg	Eqv	Pos	Neg	Pos	Neg	Eqv	Pos	Neg	Eqv	Pos	Neg
Western blot	Pos	354	85	7	401	45	2	340	105	861	6	3	861	16	7	853	12
	Neg	15	538	2	74	486	4	8	547	7	124	0	9	119	3	2	129
Sensitivity (95% CI)		80.6% (76.6%–84.2%)			89.9% (86.7%–92.5%)			76.4% (72.2%–80.3%)		99.3% (98.5%–99.7%)			98.2% (97.1%–99.0%)			98.6% (97.6%–99.3%)	
Specificity (95% CI)		97.3% (95.6%–98.5%)			86.8% (83.7%–89.5%)			98.6% (97.2%–99.4%)		94.7% (89.3%–97.8%)			93.0% (87.1%–96.7%)			98.5% (94.6%–99.8%)	

^*a*^ Equivocal (Eqv) results were not included in calculating sensitivity and specificity.

Overall, there were 16 (13 for HSV-1 IgG and 3 for HSV-2 IgG) equivocal results on the Bio-Rad instrument and 34 (18 for HSV-1 IgG and 16 for HSV-2 IgG) on the DiaSorin instrument. In contrast to the clinical testing cohort where most of the Bio-Rad results had non-equivocal final results, only 25% of the Bio-Rad equivocal results in the VRC cohort had a non-equivocal result after repeating. All four revised results became positive for HSV-1 IgG, yielding a false positive result in two cases. For DiaSorin, 53% (12 HSV-1 IgG and 6 HSV-2 IgG) of the equivocal results from this cohort had a non-equivocal final result with 11 results concordant with the WB and the remaining 7 results falsely positive for HSV-1 IgG. All equivocal samples were unique, and no samples from the VRC cohort were equivocal for multiple assays or instruments.

**Fig 4:**
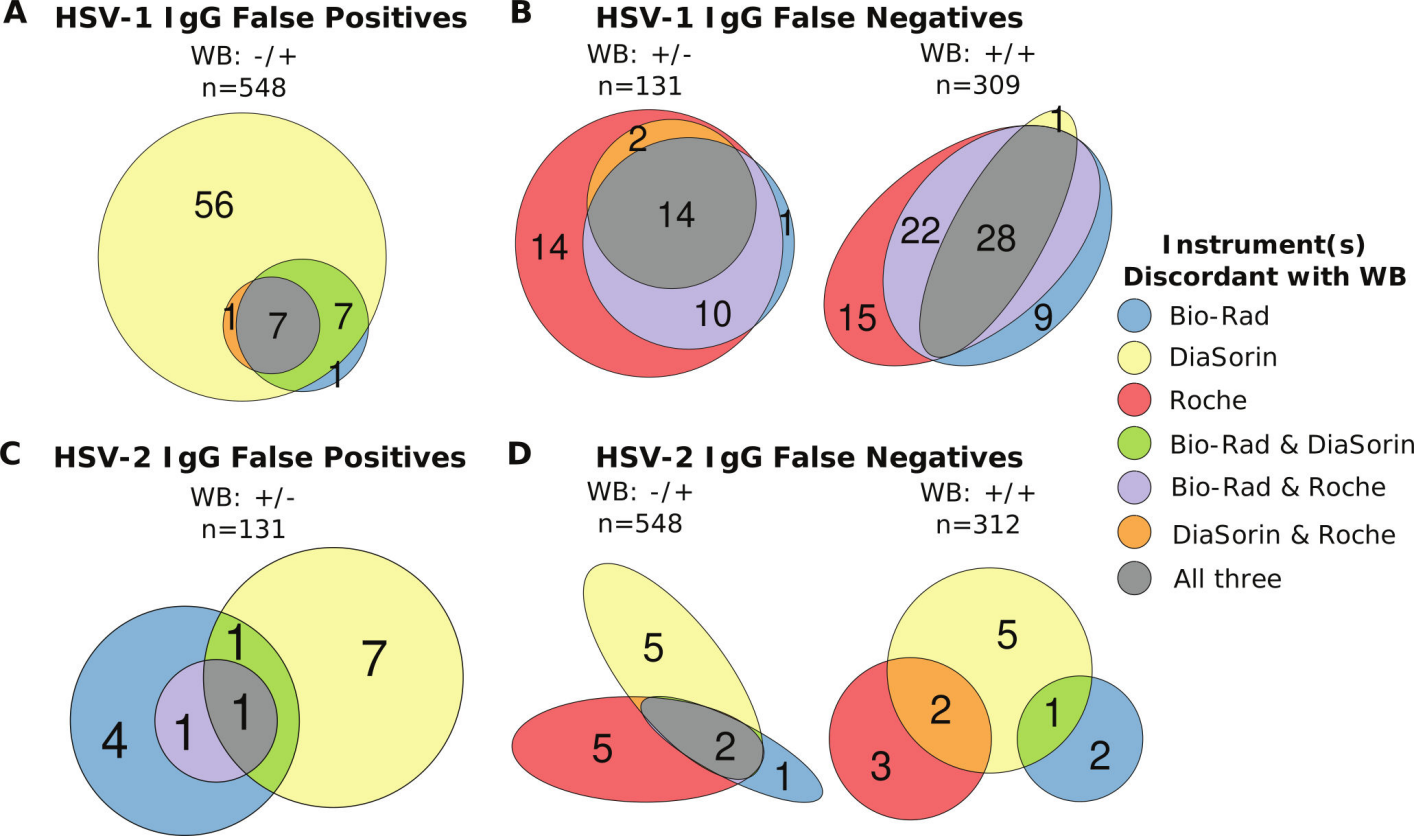
Euler plots showing the number of samples from the VRC cohort that disagree with the WB results for each automated instrument colored by which instruments were discordant with the WB results. Data are presented in Fig. 1. (**A**) Number of samples that were negative for HSV-1 IgG by WB but falsely positive on the automated instruments. (**B**) Number of samples that were positive for HSV-1 IgG by WB but falsely negative on the automated instruments. (**C**) Number of samples that were negative for HSV-2 IgG by WB but falsely positive on the automated instruments. (**D**) Number of samples that were positive for HSV-2 IgG by WB but falsely negative on the automated instruments. Plots made using the eulerr R package (16).

Patterns of discordant results for the VRC cohort were similar to those for the clinical testing cohort (Fig. 4). Overall, the DiaSorin instrument alone accounted for at least half of the total false positives for either HSV-1 IgG (78%) or HSV-2 IgG (50%). There was a trend for the VRC cohort to have a larger proportion of samples test falsely positive (13.3% for HSV-1 IgG and 10.7% for HSV-2 IgG) compared to the clinical testing cohort (10.2% for HSV-1 IgG and 6.5% for HSV-2 IgG), likely reflecting the lack of double negative samples in this cohort. There were significantly more HSV-1 IgG false negative results in the VRC cohort than in the clinical testing sample remnant cohort (25.9% vs 16.7%, *P*-value = 0.002). Conversely, there were significantly fewer HSV-2 false negative results in the VRC cohort than in the clinical testing cohort (3.1% vs 16.4%, *P*-value < 0.001).

#### Estimates of test characteristics

**Fig 5:**
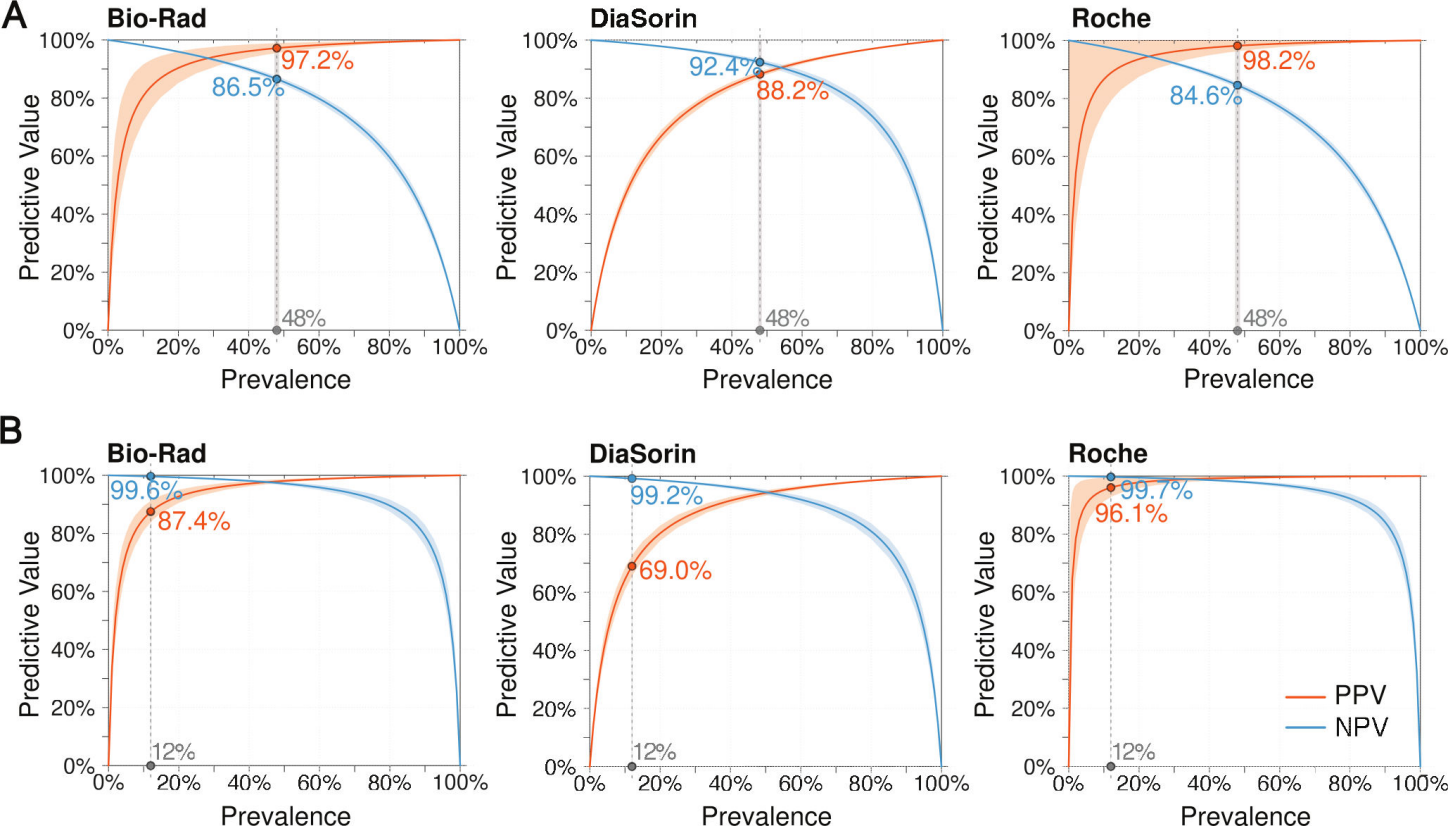
The positive and negative predictive values for each assay as a function of prevalence based on the sensitivities and specificities calculated from all samples tested. (**A**) Modeling of the positive and negative predictive values by prevalence for each HSV-1 IgG assay. The gray line indicates a population prevalence for HSV-1 in the United States of 48% (3). (**B**) Modeling of the positive and negative predictive values by prevalence for each HSV-2 IgG assay. The gray line indicates the expected population prevalence for HSV-2 in the United States of 12%. For all plots, the shaded areas represent the percent uncertainty for the sensitivity and specificity, set to the 95% CI of either the sensitivity or specificity, whichever was larger.

We calculated the sensitivities and specificities from all 1,996 samples to estimate the overall performance of these assays (Table 4). Using the 2015–2016 National Health and Nutrition Examination Survey (NHANES) population prevalence in the United States for persons aged 14–49 for HSV-1 of 48% and HSV-2 of 12% (3), we calculated the positive and negative predictive value for each assay based on the sensitivities and specificities from all samples tested (Table 4) and modeled the effect of changes in prevalence on the positive predictive value (PPV) and negative predictive value (NPV) (Fig. 5).

**TABLE 4:** Sensitivity (SN) and specificity (SP) for all instruments and all cohorts, including all samples combined

	HSV-1 IgG						HSV-2 IgG					
	Bio-Rad		DiaSorin		Roche		Bio-Rad		DiaSorin		Roche	
	SN	SP	SN	SP	SN	SP	SN	SP	SN	SP	SN	SP
All samples *n* = 1,996	83.5%	97.8%	92.1%	88.7%	80.5%	98.6%	97.1%	98.1%	94.5%	94.2%	97.9%	99.5%
Clinical testing samples *n* = 979	87.1%	98.2%	94.8%	90.4%	85.9%	98.7%	91.9%	98.8%	85.4%	94.5%	96.1%	99.7%
VRC samples *n* = 1,017	80.6%	97.3%	89.9%	86.8%	76.4%	98.6%	99.3%	94.7%	98.2%	93.0%	98.6%	98.5%

Due to the low prevalence of HSV-2, ~1 in 26 positive HSV-2 results on the Roche assay are false positives, while the likelihood that a positive HSV-2 result is a false positive is even higher for the other assays: ~1 in 8 for Bio-Rad and nearly 1 in 3 for DiaSorin. This means that if a population of 100,000 people were to be tested for HSV-2 IgG with the Roche, Bio-Rad, and DiaSorin assays, 440, 1,672, and 5,104 persons, respectively, would get false positive HSV-2 IgG results. Due to the higher prevalence of HSV-1, the positive predictive values are higher for all HSV-1 IgG assays, but the lower specificities for the HSV-1 IgG assays mean similar numbers of people (728 for Roche, 1,144 for Bio-Rad, and 5,876 for DiaSorin) would receive false positive HSV-1 IgG results if these assays were used for screening 100,000 people. The low HSV-1 NPVs also mean thousands of people (3,792 for DiaSorin to 9,360 for Roche) would test falsely negative for HSV-1 IgG if the current assays were to be used for population-based screening.

Using the semi-quantitative data, we also looked at if a different index threshold may be better for maximizing each assay’s sensitivity and specificity. Based on maximizing the receiver operating characteristic curves (Fig. S3), for the DiaSorin assays, increasing the threshold to 1.725 for HSV-1 IgG or 1.135 for HSV-2 IgG would give the optimal specificity and sensitivity. For both the Bio-Rad and Roche instruments, decreasing the threshold would yield the optimal specificity and sensitivity for the HSV-1 and HSV-2 IgG assays. This would improve overall assay accuracy, but decreasing the threshold would result in a lower specificity and more false positive results.

## DISCUSSION

In this large-scale comparison of HSV serologic diagnostic methods, we found substantial differences between the performance of three commonly used, automated FDA-cleared immunoassays for detecting HSV-1 and HSV-2 IgG manufactured by Bio-Rad, DiaSorin, and Roche. For HSV-1 IgG, there was a clear trade-off between sensitivity and specificity for all assays. The DiaSorin HSV-1 IgG assay had the highest sensitivity but the lowest specificity. On the other hand, the Bio-Rad and Roche HSV-1 IgG assays had lower sensitivities (<85%) but higher specificities (>97%). For HSV-2, there was a clear best assay with the Roche HSV-2 IgG assay having both the highest sensitivity (97.9%) and highest specificity (99.5%). The Bio-Rad HSV-2 IgG assay was nearly as good as the Roche assay, but the DiaSorin HSV-2 assay performed worst for both sensitivity and specificity. Unfortunately for laboratories, there does not seem to be a one-size-fits-all approach to automated HSV serology assays. Indeed, while our study indicates that at least some automated HSV-2 IgG assays have promising performance metrics, none of the HSV-1 IgG assays tested are adequate.

A notable aspect of our results is that we found higher specificities for HSV-2 than previous studies (14). Most prior studies compared results to enzyme-linked immunosorbent assays (ELISAs), most commonly the HerpeSelect test by Focus Diagnostics (purchased by DiaSorin in 2016). To our knowledge, this is the first large-scale comparison of a Roche HSV-1 IgG assay and the first comparison of any Roche HSV-2 IgG assay since 1999 (17). Similarly, the last large-scale comparisons of the Bio-Rad HSV-1 and HSV-2 IgG assays to other HSV serologic diagnostic methods were published in 2010–2011 (18, 19).

A strength of this study includes comparing automated HSV serology assays using samples not only measured by HSV WB but also with virologic confirmation of HSV infection. The VRC cohort provides a set of samples with a high degree of confidence in their diagnosis that serve as an excellent cohort for determining the optimal performance characteristics for these assays. Indeed, we found that the automated assays all performed quite well at detecting HSV-2 antibodies in these samples, albeit with the DiaSorin assay still having the worst performance characteristics. However, the automated assays struggled to detect HSV-1 IgG even in persons with virologically confirmed HSV-1 genital infections, potentially due to differences in serologic response to genital HSV-1, which does not recur as frequently as either genital HSV-2 or oral HSV-1 infections (20, 21). While it is encouraging to find assays that perform better than expected at detecting HSV-2 antibodies, as HSV-1 continues to increase in prevalence as a cause of genital herpes, improved serologic diagnostics are needed for both HSV-2 and HSV-1.

In December 2023, DiaSorin notified customers that current HSV-2 reagent lots, including those used in this study, may have increased numbers of equivocal or false positive results. In our study, compared to the Diasorin HSV-1 assay, the DiaSorin HSV-2 assay did not seem to be enriched for equivocal or false positive results, as the HSV-2 assay had fewer equivocal (16) or false positive (43) final results than the HSV-1 assay (21 equivocal and 133 false positive results). Of note, our data also confirms the high false positive rate of the DiaSorin HSV-2 assay seen from January to February 2021 by Quest Diagnostics Laboratory, especially for specimens with low-positive index values (22). Given the performance issues of the DiaSorin assay predate the manufacture of the lots subject to the notification, further consideration for the utility of the assay is warranted. Based on our receiver operating characteristic (ROC) analyses, even if the threshold for the DiaSorin assays were increased, the best sensitivity and specificity for their HSV-2 assay would remain worse than the other assays tested.

Limitations of this study include the use of clinical remnants rather than fresh prospectively collected specimens. Most (86%) of the VRC cohort specimens were stored frozen for >10 years, though sensitivity analysis of testing performance by date of collection demonstrated no substantial difference in assay sensitivity for samples collected prior to 2000 (*n* = 555) vs after 2000 (*n* = 462; Table S4). This study was not specifically designed to assess the effects of demographics on assay performance, but notably, we found mostly minor differences in the performance of the different assays by patient age in the clinical remnant cohort (Table S5; Supplemental Material). The VRC cohort was less diverse than the clinical remnant cohort and only included HIV-negative individuals with genital herpes, which may limit generalizability to other testing contexts. Our study also included 16 samples from persons under 18 years old, although none of the automated assays are FDA cleared for pediatric testing. Specimens were tested on the Bio-Rad instrument after one additional freeze-thaw compared to DiaSorin and Roche, and 47 of the equivocal samples were also repeated after an additional freeze-thaw, which may have slightly impacted test performance. Though the Roche assay demonstrated perfect specificity in our WB double negative cohort, only ~430 specimens were tested in this set.

Taken together, our study demonstrates that currently available HSV serologic diagnostics have limitations precluding widespread use for HSV-1 and HSV-2 testing (14, 23). Our data support the CDC’s currently recommended two-tiered algorithm for confirming HSV serological diagnoses, especially for specimens first tested with the DiaSorin assays due to their lower specificities. In our hands, the Roche HSV-2 assay performed the best compared to the WB, and overall, the Roche HSV assays would minimize potential harm from false positives as neither assay had any false positives among individuals seronegative for both HSV subtypes by WB. In addition, the Roche assay does not have equivocal results and performs well across the index value range, as opposed to the DiaSorin and Bio-Rad assays which have lower specificity at low index values and for which additional testing is required to resolve equivocal results. Overall, further work is required to adjudicate type-specific accuracy and reduce cross-reactivity among automated assays. Given the role that adsorption plays in resolving indeterminate results on the WB, it is notable that no adsorption reagents are available for automated HSV serology assays. Future work should examine how adsorption reagents could similarly resolve low-positive index values on automated assays. Further improvements and investments in HSV serological diagnostics remain necessary to support both clinical care and potential future screening.

## Data Availability

Data were analyzed using R-Studio (24). Results that remained equivocal after repeating were not included in calculations of sensitivity and specificity. All data are provided in the supplemental material.

## SUPPLEMENTAL MATERIAL

Data Set S1 jcm.00263-24-s0001.csv Line-item data for both cohorts.

Data Set S2 jcm.00263-24-s0002.xlsx Line-item data for linearity studies.

Figure S1 jcm.00263-24-s0003.pdf Linearity plot.

Figure S2 jcm.00263-24-s0004.pdf Age plot.

Figure S3 jcm.00263-24-s0005.pdf ROC curves.

Supplemental text jcm.00263-24-s0006.docx Supplemental results.

Supplemental tables jcm.00263-24-s0007.xlsx Tables S1 to S5.

ASM does not own the copyrights to Supplemental Material that may be linked to, or accessed through, an article. The authors have granted ASM a non-exclusive, world-wide license to publish the Supplemental Material files. Please contact the corresponding author directly for reuse.
