# Supplementary material for: Performance characteristics of highly automated HSV-1 and HSV-2 IgG testing
Source: J Clin Microbiol. 2024 Apr 30;62(6):e00263-24. doi: 10.1128/jcm.00263-24 (PMC11237592; doi:10.1128/jcm.00263-24)
Supplement: Figure S2 — Age plot. [file jcm.00263-24-s0004.pdf]

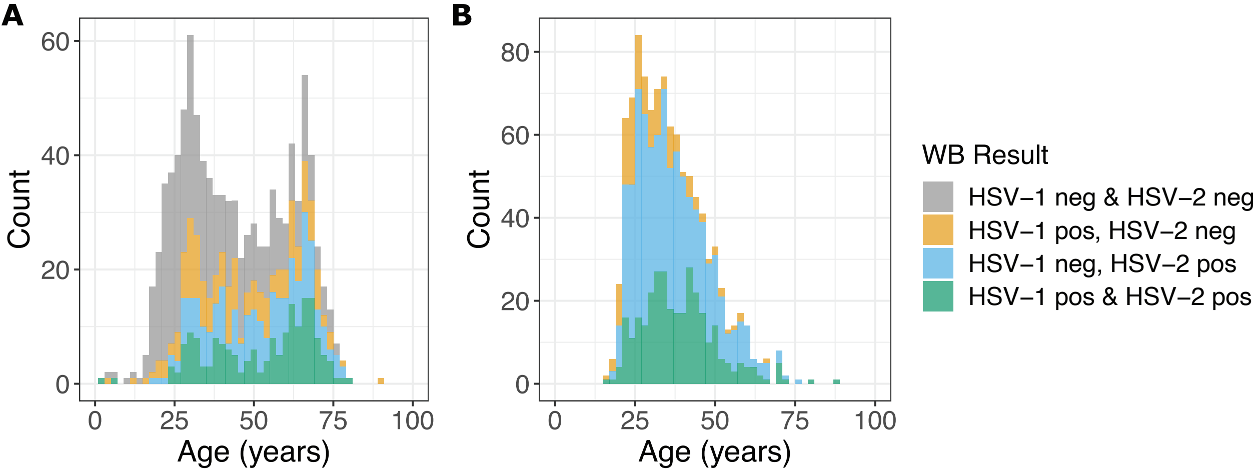

Figure S2: The age distributions for the sample cohorts. A) The clinical testing sample remnant cohort clearly shows a bimodal age distribution. As expected for serologic testing for a life-long viral infection, the number of persons positive for HSV-1 and/or HSV-2 antibodies also increases with age. B) The VRC cohort age distribution is unimodal and only the younger population is present.
